# Relative Validity and Reproducibility of a Food Frequency Questionnaire for Assessing Dietary Intakes in a Multi-Ethnic Asian Population Using 24-h Dietary Recalls and Biomarkers

**DOI:** 10.3390/nu9101059

**Published:** 2017-09-25

**Authors:** Clare Whitton, Jolene Chien Yee Ho, Zoey Tay, Salome A. Rebello, Yonghai Lu, Choon Nam Ong, Rob M. van Dam

**Affiliations:** 1 Saw Swee Hock School of Public Health, National University of Singapore and National University Health System, Singapore 117549, Singapore; ephjhcy@nus.edu.sg (J.C.Y.H.); tayzoey@nus.edu.sg (Z.T.); ephsar@nus.edu.sg (S.A.R.); ephluyng@nus.edu.sg (Y.L.); ephocn@nus.edu.sg (C.N.O.); rob.van.dam@nus.edu.sg (R.M.v.D.); 2 Department of Medicine, Yong Loo Lin School of Medicine, National University of Singapore and National University Health System, Singapore 119228, Singapore; 3 Department of Nutrition, Harvard T.H. Chan School of Public Health, Boston, MA 02115, USA

**Keywords:** food frequency questionnaire, validation, biomarkers, isoflavones, carotenoids, plasma fatty acids, 24-h dietary recall, Asia, multi-ethnic

## Abstract

The assessment of diets in multi-ethnic cosmopolitan settings is challenging. A semi-quantitative 163-item food frequency questionnaire (FFQ) was developed for the adult Singapore population, and this study aimed to assess its reproducibility and relative validity against 24-h dietary recalls (24 h DR) and biomarkers. The FFQ was administered twice within a six-month interval in 161 adults (59 Chinese, 46 Malay, and 56 Indian). Fasting plasma, overnight urine, and 24 h DR were collected after one month and five months. Intra-class correlation coefficients between the two FFQ were above 0.70 for most foods and nutrients. The median correlation coefficient between energy-adjusted deattenuated FFQ and 24 h DR nutrient intakes was 0.40 for FFQ1 and 0.39 for FFQ2, highest for calcium and iron, and lowest for energy and carbohydrates. Significant associations were observed between urinary isoflavones and soy protein intake (*r* = 0.46), serum carotenoids and fruit and vegetable intake (*r* = 0.34), plasma eicosapentaenoic acid and docosahexaenoic acid (EPA + DHA) and fish/seafood intake (*r* = 0.36), and plasma odd chain saturated fatty acids (SFA) and dairy fat intake (*r* = 0.25). Associations between plasma EPA + DHA and fish/seafood intake were consistent across ethnic groups (*r* = 0.28–0.49), while differences were observed for other associations. FFQ assessment of dietary intakes in modern cosmopolitan populations remains feasible for the purpose of ranking individuals’ dietary exposures in epidemiological studies.

## 1. Introduction

The prevalence of obesity and chronic diseases is rising rapidly in Asia [1,2]. In every Asian country, diet-related risk factors such as overweight, hypertension, and hyperglycemia are among the top contributors to early death and disability [3]. Effective interventions require a clear understanding of food consumption trends and diet-disease relationships. However, the reliable assessment of dietary intakes is increasingly challenging, as Asian diets become increasingly varied, consisting of both traditional fresh foods and ultra-processed products [4,5]. In addition, large proportions of populations reside in multi-ethnic cosmopolitan settings, where meals prepared outside the home are frequently consumed.

Singapore is one such Asian multi-ethnic cosmopolitan setting (74% Chinese, 13% Malay, 9% Indian, and 3% others) [6], with a wide variety of traditional ethnic and international cuisines, and a strong eating-out culture [7]. Long-term dietary exposures in chronic disease epidemiology are usually assessed by self-report methods such as food frequency questionnaires (FFQ). In the 1990s, a FFQ for the Singapore population was developed and validated [8]. Subsequently, new food trends have emerged, and there is growing interest in the health effects of a wider variety of nutrients. Other FFQs developed for multi-ethnic populations in the Asia region did not stratify by ethnic group in either FFQ development or validation [9,10]. Hence, a new FFQ was recently developed that incorporated ethnic stratification into the design methodology [11].

Since all dietary assessment methods based on self-reported intake are prone to some degree of measurement error, evaluating the magnitude of this error is required before use. Although there is no ‘gold standard’ for measuring dietary intakes, studies on validity relative to other dietary assessment methods can offer valuable insights. Dietary recalls or records are often used as a reference method because they are open-ended and thus do not have the same restrictions as a semi-quantitative FFQ related to a limited food list or fixed portion size. While biochemical markers are not available for all nutrients, they are also valuable as reference instruments because they are not affected by errors in recall or inaccuracies in food composition data [12]. In addition to the relative validity of an FFQ administered at a single time point, assessing the reproducibility of an FFQ over a period of time indicates the stability of the estimates of long-term dietary exposures.

This study aimed to assess the reproducibility and relative validity of a newly-developed FFQ for a multi-ethnic Asian population against two 24-h dietary recalls and nutritional biomarkers.

## 2. Materials and Methods

### 2.1. Participants and Study Design

**Figure 1:**
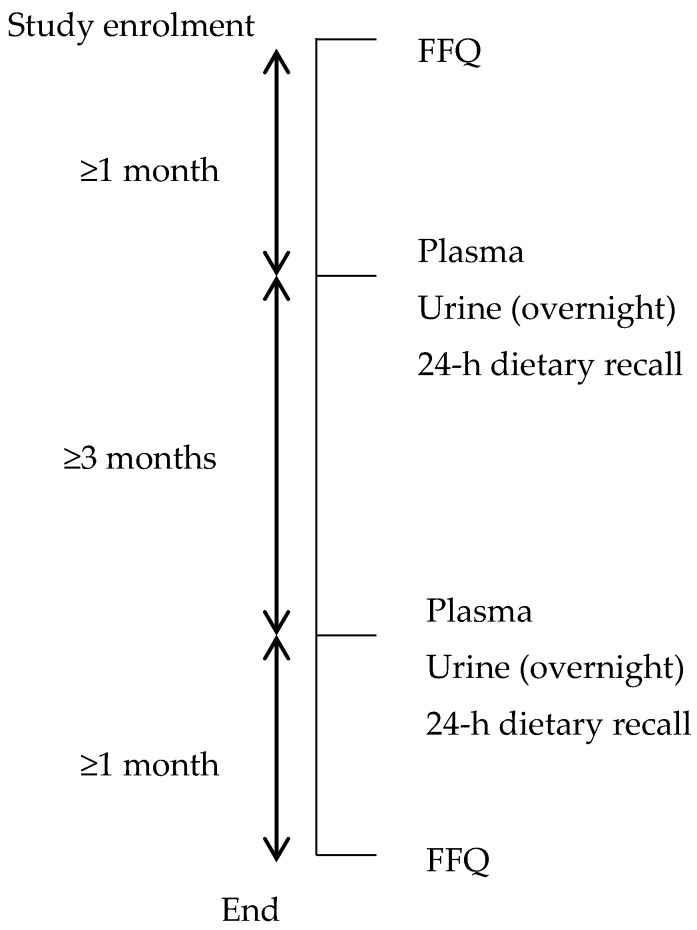
Sequence of validation study measurements.

Demographic quotas were constructed to reflect the age distribution of the adult Singapore population (aged 18–79 years), and to include equal numbers of each gender-ethnic group to enable the assessment of FFQ performance by ethnic group. We contacted participants of two population-based studies, the Singapore Population Health Study, and a national survey conducted by the Singapore Health Promotion Board. All participants had given consent to be re-contacted for future research studies. Telephone recruitment was conducted by trained interviewers. Interested participants were asked a series of screening questions to assess eligibility for the study. Participants were considered ineligible if they were residing in a household with another study participant, living in an institution, pregnant or breastfeeding, practicing/going to practice a special diet (e.g., for weight loss), unable to communicate in English or Mandarin, taking diuretic medication, diagnosed with kidney disease or a severe mental illness, or had recently changed their diet due to chronic medical conditions. We called 780 participants; 241 were uncontactable, 322 refused to participate, and 25 were ineligible. As a result, 192 participants were visited at their homes or a place of their convenience, where they provided written informed consent to take part in the study. Figure 1 summarizes the sequence of measurements. During the first study visit, the FFQ was interviewer-administered, and participants were issued with plastic screw-capped bottles (500 mL) and instructions on how to collect an overnight urine sample. During a second visit approximately one month later (median 35 days; interquartile range (IQR) 30–42 days), a fasted blood sample was drawn, filled urine bottles were collected, and a 24-h dietary recall interview was conducted. The third visit took place approximately four months after the second (median 3.75 months; IQR 3.1–4.7 months), and included the same measurements as the second visit. The fourth study visit took place approximately one month after the third (median 36 days; IQR 33–41 days) and approximately six months after the first (median 6.25 months; IQR 5.6–7.2 months). At this visit, the FFQ was interviewer-administered again, and socio-demographic information was collected. The study methodologies, protocols, and procedures were approved by the National University of Singapore Institutional Review Board (NUS IRB, reference code: B-14-082).

### 2.2. Food Frequency Questionnaire

The development of the FFQ has been described previously [11]. Briefly, in order to develop the food list, a data-driven approach was adopted, as described by Block et al. [13] using data from a nationally representative two-day 24-h dietary recall survey (*n* = 805) conducted in 2010 in adult Singapore residents aged 18–79 years. The FFQ consisted of a list of 163 food/beverage items with additional sub-questions on food sub-types and cooking methods. For each FFQ item, participants were asked how often they consumed one serving of the item, and were requested to provide the number of times either ‘per day’, ‘per week’ or ‘per month’. For items consumed less than once per month, the response category ‘Never/Rarely’ was used. Participants were asked to consider their intake over the past year when answering. For seasonal foods, interviewers converted consumption frequency during the season to an average consumption frequency over a year. A standard portion size was given for each food item, which interviewers read out for every question. Visual aids relating to the standard portion sizes were shown.

A nutrient database for the FFQ was constructed using the nationally representative 24-h dietary recall data that was used for FFQ development. Each food/drink was tagged to an FFQ line item, then data were averaged to obtain nutrient profiles for each FFQ line item that reflected the relative consumption frequencies of each food subtype covered by the line item. FFQ response data were entered into in-house data entry software. Following data extraction and cleaning, frequencies were standardized to ‘per day’ and multiplied by standard serving sizes (grams). The intake frequencies of individual fruits and vegetables were scaled up or down to align with the response to summary questions on total fruit and vegetables. For example, the intake frequency of apples was multiplied by the intake frequency of total fruit (as reported in the summary question), then divided by the sum of intake frequencies of all of the individual fruit items. All of the food intake frequencies were then merged with the FFQ nutrient database. Daily totals for energy and nutrients were calculated, followed by macronutrient intakes as a percentage of energy and micronutrient intakes (and sugar and fiber) as amounts per 1000 kcal.

### 2.3. 24-h Dietary Recall

The United States Department of Agriculture (USDA) five-step multiple-pass approach [14] was adapted to include four rather than five passes; a quick list, detailed pass, forgotten foods, and final review. In the first pass, participants were asked to list all of the foods and drinks consumed in the previous 24 h from midnight to midnight. In the second pass, the interviewer went through the list chronologically, probing for food description details, preparation methods, and amounts consumed. Visual aids, which displayed photographs of utensils, were used to assist with portion size descriptions. The third pass attempted to elicit any forgotten items such as condiments or beverages. The fourth pass reviewed all of the information. The USDA’s ‘Time and occasion’ step was omitted because it was found to be redundant in pre-tests, as the information was provided either spontaneously during the first pass, or in response to probing in the second pass. Interviewers aimed to conduct one recall on a working day and the other on a non-working day, to account for intra-individual variation between day type. Interviews were audio recorded for quality control purposes. Data were entered into in-house software that contained the Singapore Health Promotion Board’s food composition database [15].

### 2.4. Biological Samples

Participants were twice instructed to collect an overnight urine sample that included the first morning urine and any urine passed during the night. At the start of the study visit, participants were asked if their urine corresponded to the required collection period. If it did not, new urine bottles were issued and the visit was rearranged. Fasting venous blood was drawn and collected in ethylenediaminetetraacetic acid (EDTA) tubes and plain tubes. Both blood and urine samples were stored at −4 °C for a maximum of 24 h before processing, aliquoting into 1 mL tubes, and freezing at −80 °C. Blood was centrifuged to separate the plasma, and ascorbic acid preservative 2% was added to urine samples before freezing. Each participant’s two plasma/urine samples collected at the two time points (i.e., one month and five months) were analyzed pairwise in the same batch to minimize the effect of inter-batch variation on biomarker measurements. For each analyte and each batch (which corresponded to one day of laboratory measurements), split blinded quality control samples were used to assess the coefficient of variation within and between batches.

High performance liquid chromatography (HPLC) [16] was used to measure plasma lipophilic antioxidants, including lutein, zeaxanthin, α and β-cryptoxanthin, α and β-carotene, lycopene, and retinol. Plasma concentrations were quantified using photodiode array detection. The within-day and between-day coefficients of variation (CVs) were as follows: lutein (6.5 and 25.0%), zeaxanthin (6.9 and 21.3%), α-cryptoxanthin (5.5 and 18.1%), β-cryptoxanthin (4.3 and 12.2%), α-carotene (8.2 and 15.1%), β-carotene (6.1 and 12.2%), lycopene (8.6 and 14.9%), and retinol (4.3 and 10.8%).

The concentration of 19 plasma fatty acids was measured using gas chromatography-tandem mass spectrometry conducted on an Agilent 7890 GC system equipped with a 7001B Triple Quadrupole mass detector (Agilent, Santa Clara, CA, USA) [17]. Both free and esterified (TGs, phospholipids, cholesterol esters) fractions were measured. The within-day and between-day CVs were as follows: pentadecylic acid (3.2 and 12.1%), margaric acid (2.6 and 15.7%), linoleic acid (2.8 and 16.4%), γ-linolenic acid (3.4 and 15.2%), α-linolenic acid (ALA) (2.7 and 11.4%), eicosadienoic acid (3.8 and 11.1%), dihomo-γ-linolenic acid (2.9 and 9.9%), arachidonic acid (2.3 and 13.7%), eicosatrienoic acid (6.4 and 25.1%), eicosapentaenoic acid (EPA) (2.7 and 15.8%), and docosahexaenoic acid (DHA) (3.3 and 9.1%).

In order to evaluate the FFQ’s ability to assess soy intake, the concentration of urinary metabolites daidzein, glycitein, genistein, and equol were measured using a HPLC ( Dionex UltiMate 3000 LC system, Thermo Fisher Scientific, Waltham, MA, USA) from an established LC-MS/MS method [18] using phenyl C6 chromatography coupled with photodiode array and fluorometric detections. The within-day and between-day CVs were as follows: daidzein (4.0 and 17.0%), equol (17.9 and 30.2%), glycitein (8.4 and 18.4%), and genistein (4.5 and 12.7%). Urinary creatinine was measured using the Jaffe method (reaction with alkaline picrate using an auto-analyzer). Urinary metabolite concentrations were expressed as nmol/mg creatinine.

### 2.5. Statistical Analysis

Correlation coefficients in the range of 0.4–0.6 have been reported between FFQ nutrient intake estimates and reference instruments [12]. To estimate the sample size required to distinguish between these values, the formula:

[*n* = (Z_α_ + Z_β_)^2^ σ^2^/d^2^]

was used, with Fisher’s Z transformation of correlation coefficients, where σ^2^ = 1 for the Z-scale [19]. For α = 0.05 and (1 − β) = 0.80 (i.e., 80% power), the number of required participants was 110. Within-person variation in the reference instrument indicates the need for a larger sample size [12]; therefore, we estimated *n* = 150 to be the minimum number of participants required. To account for an expected 20% attrition rate, we increased the target sample size to *n* = 192.

Dietary recall data were checked for errors by examining outlying values. Weightings of 5.5/7 were applied to work days, and 1.5/7 to non-working days to reflect typical work patterns, before calculating average daily intakes. Macronutrients for both FFQ and dietary recall data were expressed as a percentage of total energy (%E), while micronutrients were expressed as amount per 1000 kcals. Both energy and nutrient variables were transformed using natural logs to obtain a normal distribution. For food groups, 1 g was added to remove zeros before applying transformations. Either natural log or square root transformations were used in analyses depending on which best improved normality.

Plasma polyunsaturated fatty acids (PUFA), odd-chain saturated fatty acids (SFA) and EPA + DHA as a percentage of total plasma fatty acids (% total FA) were calculated, in order to evaluate the relative validity of the FFQ in assessing PUFA intake, dairy fat intake [20], and fish and seafood intake [21], respectively. Values were transformed using natural logs. All of the values were within four standard deviations of the mean. The mean concentration of analytes from the two time points were used in subsequent analyses.

Descriptive statistics were calculated where ANOVA was used to compare continuous variables, and chi-squared was used to compare categorical variables. Pearson correlation coefficients were calculated to examine the associations between FFQ and dietary recall nutrient intakes, and between selected FFQ measures and urinary isoflavones, serum carotenoids, and plasma fatty acids. Users of carotenoid-containing supplements were excluded from analyses involving serum carotenoids, and users of phytoestrogen-containing supplements were excluded from analyses involving urinary isoflavones, because the supplement contents could not be quantified. Partial correlations were calculated, and then adjusted for ethnicity, age, sex, energy intake, and additionally total fat (as a percentage of energy intake) for carotenoid associations only. Values were adjusted for intra-individual variation using the intra-class correlation coefficient (ICC) and adopting the formula described by Rosner and Willett [22]. The ICCs between the two time points were calculated by ANOVA for biomarker concentrations and 24-h dietary recalls (using transformed energy-adjusted nutrient intakes) in order to deattenuate the correlation coefficients for intra-individual variation. The ICCs between the two time points were also calculated for the FFQ to examine reproducibility. IBM SPSS version 23 (IBM Corp, Armonk, NY, USA) was used for statistical analyses, with the level of significance set at 5%.

## 3. Results

Even distributions of ethnicity, gender and age groups were recruited, and most participants had education beyond secondary school level (Table 1). Of the 192 participants enrolled, 31 (16%) dropped out (6 Chinese, 8 Indians, and 17 Malays), and 161 completed the study.

The ICC of the FFQ estimates, a measure of reproducibility, was above 0.70 for most foods and nutrients (Table 2), and values were similar among the ethnic groups except in Malays, where the ICCs for vitamin A, vitamin C, and fruit intake were below 0.40.

The median correlation coefficient for the association between FFQ and 24-h dietary recall nutrient intakes was 0.40 for FFQ1 and 0.39 for FFQ2. Associations of the FFQ protein (*r* = 0.45–0.53) and total fat intakes (*r* = 0.35–0.39) as a percentage of energy were higher than for carbohydrates (*r* = 0.20–0.25) (Table 3). For PUFA, there was a stronger association between FFQ2 and dietary recall values (*r* = 0.31) as compared with FFQ1 (*r* = 0.09). The magnitude of correlations for other nutrients was similar for FFQ1 and FFQ2. The highest correlation coefficients were observed for micronutrients, for example, between the FFQ and dietary recall values for calcium (*r* = 0.57–0.68) and iron (*r* = 0.50–0.64).

Table 4 shows correlations between FFQ intake estimates and related biomarkers. Significant associations were observed between urinary isoflavone concentration and FFQ soy protein intake, serum total carotenoid concentration and FFQ fruit and vegetable intake, plasma EPA+DHA concentration and FFQ fish/seafood intake, and plasma odd chain SFA and FFQ dairy fat intake. Correlations for dairy fat, fruit, and soy protein with relevant biomarkers were higher for FFQ2 than for FFQ1, whereas correlations for fish/seafood were higher for FFQ1. Adjustments for demographic factors and correction for within-person variation in biomarkers did not substantially change the observed associations. The exclusion of fish oil supplement users did not affect the magnitude of associations (data not shown). Based on FFQ2, the weakest correlation was observed between intake and plasma levels of PUFA (*r* = 0.12), and the highest correlations were found between fish/seafood and plasma EPA + DHA (*r* = 0.36), and soy protein and isoflavones (*r* = 0.46).

Stratified analyses highlighted differences between ethnic groups for correlations between FFQ estimated intakes and biomarkers (Table 5 and Supplementary Table S1). For instance, fruit and vegetable associations with total carotenoid concentration and with individual carotenoids was present among Chinese participants (from *r* = 0.30 for lutein, to *r* = 0.52 for alpha-carotene) and to some extent Malays although, besides lycopene, associations did not reach statistical significance, but were absent among Indians. There was some indication of an association between curry gravy and total carotenoid concentration in Chinese (*r* = 0.19) and Malays (*r* = 0.12), but this was not statistically significant (data not shown). The association between soy protein intake and urinary isoflavones was present only among Malays (*r* = 0.58) and Indians (*r* = 0.64), but was not significant among Chinese (*r* = 0.31). The association between plasma EPA + DHA concentrations with fish/seafood intake was consistent across ethnic groups (*r* = 0.28–0.49). Differences were also noted between males and females, with a stronger correlation for fruit and serum carotenoids in women and a stronger correlation for dairy fat and plasma odd chain SFA in men (Supplementary Table S2).

## 4. Discussion

The purpose of this study was to assess the reproducibility and relative validity of a new multi-ethnic FFQ in an urban Asian setting, using repeat FFQ administrations to assess reproducibility and 24-h dietary recalls and plasma and urine concentration biomarkers to assess relative validity. Our results suggest reasonable accuracy and good reproducibility for evaluated FFQ assessments of dietary intakes.

In comparison with reproducibility studies of other FFQs, in which values ranged from 0.26–0.91 [23,24,25], we observed relatively high ICCs, which indicated the good reproducibility of our FFQ assessment. This implies that a single FFQ is likely to be sufficient to capture habitual dietary intake, although repeated FFQ administration may be required to accurately reflect dietary changes for cohort studies with long follow-up periods. It should be noted, however, that the timeframe of six months used in this study was shorter than that of other studies, which often used a 12-month interval. Although a six-month timeframe can introduce seasonal variation, such variation is minimal in Singapore for most foods. However, there may have been some effect related to various cultural celebrations that take place throughout the year, which may have resulted in minor attenuation of ICCs. Poorer reproducibility was observed in Malays for vitamins A and C, which may be explained by the low reproducibility for fruit intake. This could be related to some seasonal variation for fruit intake such as durian, or because of the Ramadan fasting month, which took place during the study period, as ethnic Malays are generally Muslims, and changes in dietary intakes have been observed [26].

Despite soy intakes in Singapore being lower than in some other Asian populations, such as Japan and parts of China [27], the strength of association observed between soy protein intake and urinary isoflavones (*r* = 0.46) was of a similar magnitude to that observed in these populations, for example in Shanghai men (*r* = 0.48) [28] and Japanese adults (*r* = 0.30–0.40) [29]. However, results may not be directly comparable, since we used soy protein intake as a proxy for total isoflavone intake, while other studies estimated the isoflavone contents of FFQ items. Although, with the limitation of wide variability in the isoflavone content of foods [30], it is not clear which approach is superior, as demonstrated in populations such as the United States, where both approaches have been used [31,32,33,34]. The relative validity of our FFQ in assessing fish and seafood intake, as indicated by the association with plasma EPA + DHA, was comparable to observations in other studies [21,35]. The associations between dairy fat intake and odd-chain SFAs were also comparable to the results of other studies [36,37]. The stronger association in males as compared with females, and Chinese and Indians as compared with Malays may be explained by differences in data capture and/or reporting accuracy by dairy product type. For instance, in Malays, condensed milk consumed in beverages was the top contributor (22%) to total dairy fat intake, whereas this item only contributed to 11% (Chinese) and 12% (Indian) of dairy fat intake in other groups (data not shown). In females, ice cream was the top contributor to dairy fat intake (22%, as compared with 13% in males). It may be that the intake frequency of ice cream is less well recalled than the intake frequency of milk, for example. On the other hand, it may be that a sub-type question on whether the ice cream was low fat would have allowed a more accurate assignment of a fat value, and thus would have improved the correlation.

The magnitude of associations between serum carotenoids and FFQ fruit and vegetable intake are consistent with results observed in other studies [38,39]. However, substantial differences were observed between ethnic groups in our study, with high correlations in Chinese and the absence of association between FFQ fruit and vegetable intake and individual and total carotenoids in Indians. This lack of association in Indians is difficult to explain. It may be related to co-ingestion of carotenoid absorption enhancers such as fat or inhibitors such as fiber [12]. Fiber intake was significantly higher in Indians as compared with other ethnic groups. The result could also be related to the types of fruit and vegetables consumed by this group, although data on intakes did not indicate as such. Another possible explanation is the consumption of other carotenoid-containing foods by this group such as powdered spices or food colourants. Although no association between curry gravies and serum carotenoids was observed in Indians, this item represents only a fraction of the foods that potentially contain carotenoids. Cooking method may also play a role in these observations because of its effect on carotenoid bioavailability [12]. Another explanation could be related to the assessment of portion sizes for specific fruits and vegetables consumed by this group. Generally, the second FFQ performed better than the first FFQ, which is likely to be related to the matching timeframe of FFQ with the biomarker measurement.

With the exception of weak correlations for carbohydrate and energy intakes, the relative validity of the FFQ in assessing nutrient intakes compared with the repeat 24-h dietary recalls was similar to results observed in other studies in Asia [23,40], and in a multi-ethnic Western population [41]. It is unclear why carbohydrate was less well assessed by the FFQ in comparison to nutrients, but may be related to portion size assessment. A study in China using portion size photographs along with an FFQ reported a correlation coefficient for carbohydrate of *r* = 0.53 between FFQ estimates and repeat 24-h dietary recalls [23]. The portion sizes used in this study were based on a combination of standard serving sizes, national level intake data, and weighed samples from food outlets. For example, the portion size used for plain rice was 1 rice bowl (200 g), the amount commonly served in food outlets. Although eating out is common, with 60% of Singapore residents eating out at least four times per week [7], serving sizes of rice at home may be variable. As other studies have indicated that collecting information on portion size does not necessarily improve FFQ estimates [42,43,44], the FFQ was not designed to collect this information. However, the importance of portion size assessment may warrant further investigation in this population.

The strengths of this study include the diverse sample size and the collection of repeat reference measurements covering a range of dietary components. FFQ and 24-h dietary recall share some biases, such as the use of food composition data, dependency on participants recall, and susceptibility to desirability bias. Biological markers represent a more independent measure, although they too are not a ‘gold standard’ since they are affected by varying bioavailability. The biomarkers used were concentration biomarkers, and we did not use biomarkers that assess absolute intakes such as 24-h urinary nitrogen for study feasibility reasons, the implication being a reliance on the two-day 24-h dietary recall data to evaluate FFQ estimates for most nutrients. Smoking [45,46] and body weight status [47] may modulate serum carotenoid concentrations, but information on these factors was not collected in this study. We would expect stronger correlations between dietary fruit/vegetable consumption and carotenoids than what we observed had we accounted for smoking and body weight. Due to budget constraints, our sample size within each ethnic group was limited, meaning that some of our analyses may have been underpowered, and more weight should be placed on analyses of the whole sample. Another limitation that may have attenuated the observed correlation coefficients was the absence of data on the carotenoid content of individual foods. This results in a loss of precision due to the varying contents of various carotenoids in different fruits and vegetables; for example, some participants may have consumed a high intake of fruit, but only items with low carotenoid content. We noted good reproducibility of biomarker measurements apart from isoflavones, but poor reproducibility of all nutrients assessed by the 24-h dietary recall, and extremely poor when analyses were stratified by ethnic group, particularly in Malay participants (data not shown). This suggests that more than two repeats of the 24-h dietary recall may be required to obtain a better estimate of habitual intake using this measure, and two days of data was not a suitable reference method for all nutrients.

## 5. Conclusions

This study evaluated the relative validity and reproducibility of a 163-item semi-quantitative FFQ developed specifically for a multi-ethnic Asian population. The results showed that the FFQ performed similarly to other FFQs in the literature for most nutrients, although we identified exceptions for specific dietary intakes in certain ethnic groups. The Singaporean population is characterized by the consumption of a wide variety of foods from different Asian and international cuisines and a high frequency of eating out. Our results suggest that the assessment of dietary intakes in such modern cosmopolitan populations remains feasible for the purpose of ranking individuals’ dietary exposures in epidemiological studies.

## Figures and Tables

**Table 1:** Study participant characteristics and intakes of selected foods and nutrients.

Characteristics	Total	Chinese	Malay	Indian	
	*n* = 161	*n* = 59	*n* = 46	*n* = 56	*p*
Age (years)	44 ± 14	44 ± 16	43 ± 13	43 ± 14	0.9
Sex (% male)	50%	51%	50%	50%	0.995
*Housing type*					0.945
1–3 room government flat	39%	39%	37%	39%	
4–5 room government flat	58%	56%	61%	57%	
Private flat or landed property	4%	5%	2%	4%	
*Monthly household income (S$)*					0.052
<2000	20%	21%	20%	17%	
2000–3999	32%	25%	43%	30%	
4000–5999	17%	17%	10%	24%	
6000–9999	20%	15%	28%	17%	
≥10,000	12%	21%	0%	11%	
*Highest educational attainment*					0.019
Primary or below	11%	15%	11%	7%	
Secondary	11%	8%	11%	14%	
Higher education inc. vocational	53%	44%	71%	46%	
University	25%	32%	7%	32%	
Carotenoid supplement user	16%	14%	24%	11%	0.163
Phytoestrogen supplement user	15%	14%	24%	9%	0.1
*Biomarker concentrations*					
Isoflavones (μg/gUCr)	599 ± 868	757 ± 925	518 ± 1040	500 ± 601	0.036
Total carotenoids (mg/L plasma)	1.48 ± 0.43	1.66 ± 0.54	1.41 ± 0.34	1.35 ± 0.27	0.001
Plasma EPA + DHA (% total FA)	2.08 ± 0.98	2.33 ± 1.03	2.12 ± 1.09	1.78 ± 0.74	0.002
Plasma PUFA (% total FA)	56 ± 4	56 ± 4	55 ± 4	56 ± 5	0.2
Plasma odd chain SFA (% total FA)	0.16 ± 0.04	0.16 ± 0.04	0.14 ± 0.03	0.17 ± 0.04	0.005
*Food and nutrient intakes* ^1^					
Protein (%E)	14 ± 2	16 ± 2	14 ± 2	13 ± 2	<0.001
Carbohydrate (%E)	50 ± 6	46 ± 7	50 ± 5	53 ± 5	<0.001
Total fat (%E)	35 ± 5	35 ± 5	36 ± 4	33 ± 5	0.02
Saturated fat (%E)	13 ± 3	13 ± 2	14 ± 2	13 ± 3	0.004
Monounsaturated fat (%E)	13 ± 3	14 ± 3	13 ± 2	13 ± 3	0.049
Polyunsaturated fat (%E)	6 ± 2	7 ± 2	6 ± 2	6 ± 2	0.2
Sugar (g/1000 kcal)	38 ± 10	35 ± 10	41 ± 10	40 ± 11	0.005
Fibre (g/1000 kcal)	9 ± 2	9 ± 2	8 ± 1	10 ± 2	<0.001
Vitamin A (RE, mcg/1000 kcal)	358 ± 93	371 ± 108	347 ± 64	355 ± 94	0.7
Vitamin C (mg/1000 kcal)	51 ± 21	54 ± 24	48 ± 16	51 ± 21	0.7
Calcium (mg/1000 kcal)	305 ± 86	296 ± 76	288 ± 69	327 ± 103	0.07
Iron (mg/1000 kcal)	6.9 ± 1.2	7.1 ± 1.2	6.6 ± 1.2	7.0 ± 1.2	0.041
Energy (kcal/day)	2679 ± 1036	2609 ± 1006	2883 ± 1201	2586 ± 911	0.4
Fruit (inc. 100% fruit juice) (g/day)	161 ± 101	174 ± 114	143 ± 84	163 ± 100	0.4
Vegetables (g/day)	83 ± 54	101 ± 65	71 ± 41	74 ± 45	0.004
Dairy fat (g/day)	5 ± 5	4 ± 4	7 ± 6	6 ± 4	0.001
Soy protein (g/day)	3 ± 3	3 ± 2	3 ± 3	3 ± 3	0.1
Fish/seafood (g/day)	41 ± 31	47 ± 31	54 ± 34	25 ± 18	<0.001

EPA, eicosapentaenoic acid; DHA, docosahexaenoic acid; FA, fatty acid; PUFA, polyunsaturated fatty acid; SFA, saturated fatty acid; %E, as a percentage of energy intake; RE, retinol equivalents; inc., including. Values are percentages for categorical variables, and means ± SD for continuous variables. ^1^ Food and nutrient intake values are the mean of the two food frequency questionnaires (FFQs).

**Table 2:** Reproducibility (intra-class correlation coefficients) for repeat FFQ administrations with a six-month interval, in the total group and according to ethnicity.

Nutrient/Food	Total	Chinese	Malay	Indian
	*n* = 161	*n* = 59	*n* = 46	*n* = 56
Energy (kcal/day)	0.78 (0.69, 0.85)	0.83 (0.71, 0.90)	0.72 (0.44, 0.85)	0.80 (0.62, 0.89)
Protein (%E)	0.78 (0.70, 0.84)	0.73 (0.55, 0.84)	0.62 (0.31, 0.79)	0.62 (0.35, 0.78)
Total fat (%E)	0.70 (0.59, 0.78)	0.73 (0.54, 0.84)	0.70 (0.45, 0.83)	0.65 (0.40, 0.79)
Saturated fat (%E)	0.77 (0.69, 0.83)	0.68 (0.46, 0.81)	0.78 (0.60, 0.88)	0.80 (0.65, 0.88)
Monounsaturated fat (%E)	0.69 (0.58, 0.78)	0.71 (0.51, 0.82)	0.72 (0.49, 0.84)	0.66 (0.42, 0.80)
Polyunsaturated fat (%E)	0.77 (0.68, 0.83)	0.73 (0.55, 0.84)	0.81 (0.65, 0.89)	0.77 (0.61, 0.86)
Carbohydrate (%E)	0.74 (0.65, 0.81)	0.66 (0.43, 0.80)	0.76 (0.56, 0.87)	0.68 (0.46, 0.81)
Sugar (g/1000 kcal)	0.70 (0.59, 0.78)	0.74 (0.57, 0.85)	0.65 (0.37, 0.81)	0.62 (0.36, 0.78)
Fiber (g/1000 kcal)	0.85 (0.79, 0.89)	0.82 (0.71, 0.90)	0.72 (0.50, 0.85)	0.87 (0.79, 0.93)
Vitamin A (mcg/1000 kcal)	0.67 (0.55, 0.76)	0.79 (0.65, 0.88)	0.35 (−0.16, 0.63)	0.64 (0.38, 0.79)
Vitamin C (mg/1000 kcal)	0.66 (0.53, 0.75)	0.76 (0.59, 0.86)	0.37 (−0.12, 0.64)	0.69 (0.47, 0.82)
Calcium (mg/1000 kcal)	0.75 (0.66, 0.82)	0.77 (0.62, 0.86)	0.64 (0.36, 0.80)	0.78 (0.62, 0.87)
Iron (mg/1000 kcal)	0.75 (0.66, 0.82)	0.70 (0.50, 0.82)	0.74 (0.52, 0.85)	0.79 (0.65, 0.88)
Fruit (inc 100% fruit juice) (g/day)	0.62 (0.47, 0.72)	0.86 (0.77, 0.92)	0.22 (−0.43, 0.57)	0.52 (0.18, 0.72)
Vegetables (g/day)	0.62 (0.48, 0.72)	0.51 (0.19, 0.71)	0.64 (0.35, 0.80)	0.68 (0.46, 0.81)
Dairy fat (g/day)	0.83 (0.76, 0.87)	0.85 (0.75, 0.91)	0.74 (0.52, 0.85)	0.85 (0.74, 0.91)
Soy protein (g/day)	0.71 (0.61, 0.79)	0.70 (0.49, 0.82)	0.79 (0.62, 0.88)	0.64 (0.38, 0.79)
Fish/seafood (g/day)	0.80 (0.73, 0.86)	0.87 (0.77, 0.92)	0.66 (0.40, 0.81)	0.77 (0.60, 0.86)

%E, as a percentage of energy. Values are intra-class correlation coefficients (95% confidence interval, or CI). Energy and nutrients were transformed using natural logs. Foods (*x* + 1) were square-root-transformed.

**Table 3:** Pearson correlations between FFQ1 and FFQ2 with 24-h dietary recalls, *n* = 161.

Nutrient	FFQ1			FFQ2		
	Crude	Adjusted ^1^	Deatt. ^2^	Crude	Adjusted ^1^	Deatt. ^2^
Protein (%E)	0.39	0.33	0.53 *	0.36	0.28	0.45 *
Total fat (%E)	0.30	0.26	0.35 *	0.33	0.30	0.39 *
Saturated fat (%E)	0.35	0.33	0.48 *	0.27	0.26	0.38 *
Monounsaturated fat (%E)	0.28	0.24	0.39 *	0.23	0.20	0.32 *
Polyunsaturated fat (%E)	0.04	0.04	0.09	0.16	0.15	0.31
Carbohydrate (%E)	0.28	0.19	0.25 *	0.25	0.15	0.20
Sugar (g/1000 kcal)	0.33	0.31	0.39 *	0.32	0.30	0.39 *
Fiber (g/1000 kcal)	0.47	0.41	0.49 *	0.51	0.47	0.56 *
Vitamin A (mcg/1000 kcal)	0.11	0.21	0.40 *	0.10	0.17	0.32 *
Vitamin C (mg/1000 kcal)	0.36	0.37	0.50 *	0.31	0.32	0.43 *
Calcium (mg/1000 kcal)	0.40	0.38	0.68 *	0.34	0.32	0.57 *
Iron (mg/1000 kcal)	0.36	0.33	0.50 *	0.43	0.41	0.64 *
Energy (kcal/day)	0.15	0.03	0.04	0.11	0.02	0.02

Deatt., deattenuated; %E, as a percentage of energy. ^1^ Adjusted for ethnicity, age, and sex. ^2^ Adjusted for ethnicity, age, and sex, and corrected for intra-individual variation between two 24-h dietary recalls. * Correlation is statistically significant, *p* < 0.05.

**Table 4:** Pearson correlations between FFQ1 and FFQ2, and biomarkers, *n* = 161.

Biomarker and FFQ Measurement	FFQ1			FFQ2		
	Crude	Adjusted ^1^	Deatt. ^2^	Crude	Adjusted ^1^	Deatt. ^2^
Isoflavones (μg/gUCr)						
Soy protein (g/day) ^3^	0.21	0.20	0.27 *	0.31	0.32	0.46 *
Total carotenoids (mg/L plasma)						
Fruit (inc 100% fruit juice) (g/day) ^4^	0.12	0.14	0.15	0.29	0.30	0.31 *
Vegetables (g/day) ^4^	0.25	0.18	0.19 *	0.20	0.21	0.22 *
Total fruit and vegetables (g/day) ^4^	0.20	0.19	0.20 *	0.31	0.33	0.34 *
Plasma EPA + DHA (% total FA)						
Fish/seafood (g/day)	0.47	0.48	0.51 *	0.34	0.35	0.36 *
Plasma polyunsaturated FA (% total FA)						
Polyunsaturated fat (%E)	0.12	0.14	0.15	0.14	0.12	0.12
Plasma odd chain saturated FA (% total FA)						
Dairy fat (g/day)	0.11	0.14	0.15	0.18	0.24	0.25 *

Deatt., deattenuated; EPA, eicosapentaenoic acid; DHA, docosahexaenoic acid; %E, as a percentage of energy intake; FA, fatty acid. Values were transformed using natural logs or square roots before analysis. ^1^ Adjusted for energy intake, ethnicity, age, and sex. Carotenoid associations additionally adjusted for total fat intake (as a % of energy). ^2^ Adjusted for energy intake, ethnicity, age, and sex, and corrected for intra-individual variation between two biomarker measurements. Carotenoid associations additionally adjusted for total fat intake (as a % of energy). ^3^ Phytoestrogen supplement users were excluded (FFQ1 *n* = 18; FFQ2 *n* = 17). ^4^ Carotenoid supplement users were excluded (FFQ1 *n* = 18; FFQ2 *n* = 17). * Correlation is statistically significant, *p* < 0.05.

**Table 5:** Pearson correlations between FFQ2 and biomarkers, by ethnic group, *n* = 161.

Biomarker and FFQ Measurement	Chinese	Malay	Indian
	*n* = 59	*n* = 46	*n* = 56
Isoflavones (μg/gUCr)			
Soy protein (g/day) ^1^	0.31	0.58 *	0.64 *
Total carotenoids (mg/L plasma)			
Fruit (inc 100% fruit juice) (g/day) ^2^	0.41 *	0.13	0.11
Vegetables (g/day) ^2^	0.29 *	0.28	−0.15
Total fruit and vegetables (g/day)^2^	0.43 *	0.22	0.04
Plasma EPA + DHA (% total FA)			
Fish and seafood (g/day)	0.35 *	0.49 *	0.28 *
Plasma polyunsaturated FA (% total FA)			
Polyunsaturated fat (%E)	0.08	0.02	0.24
Plasma odd chain saturated FA (% total FA)			
Dairy fat (g/day)	0.36 *	0.13	0.37 *

EPA, eicosapentaenoic acid; DHA, docosahexaenoic acid; %E, as a percentage of energy intake; FA, fatty acid. Values were transformed using natural logs or square roots before analysis. Values were adjusted for intra-individual variation, age, sex, and energy intake. Carotenoid associations additionally adjusted for total fat intake (as a % of energy). ^1^ Phytoestrogen supplement users were excluded (*n* = 17). ^2^ Carotenoid supplement users were excluded (*n* = 17). * Correlation is statistically significant, *p* < 0.05.

## Supplementary Materials

The following are available online at www.mdpi.com/2072-6643/9/10/1059/s1, Table S1: Pearson correlations between FFQ2 total fruit and vegetables and individual serum carotenoid concentrations, *n* = 144; Table S2: Pearson correlations between FFQ2 and biomarkers, by sex.
