# Supplementary material for: Relative Validity and Reproducibility of a Food Frequency Questionnaire for Assessing Dietary Intakes in a Multi-Ethnic Asian Population Using 24-h Dietary Recalls and Biomarkers
Source: Nutrients. 2017 Sep 25;9(10):1059. doi: 10.3390/nu9101059 (PMC5691676; doi:10.3390/nu9101059)
Supplement: Supplementary file 1 [file nutrients-09-01059-s001.zip › nutrients-224445-supplementary.pdf]

Table S1. Pearson correlations between FFQ2 total fruit and vegetables and individual serum carotenoid concentrations, n = 144[1]

Carotenoid (mg/L plasma)	All[2] n = 144	Chinese[3] n = 53	Malay[3] n = 39	Indian[3] n = 52
Alpha-carotene	0.24*	0.52*	0.14	-0.07
Beta-carotene	0.19*	0.47*	0.17	-0.20
Alpha-cryptoxanthin	0.12	0.39*	0.18	-0.31
Beta-cryptoxanthin	0.25*	0.44*	0.25	-0.12
Lycopene	0.21*	0.32*	0.41*	0.01
Lutein	0.23*	0.30*	0.23	0.10
Zeaxanthin	0.05	0.15	0.24	-0.17

Values were transformed using natural logs or square roots before analysis

[1] Carotenoid supplement users were excluded

[2] Adjusted for intra-individual variation, ethnicity, age, sex, fat intake (% energy), and energy intake

[3] Adjusted for intra-individual variation, age, sex, fat intake (% energy), and energy intake

* Correlation is statistically significant, $P < 0.05$

Table S2. Pearson correlations between FFQ2 and biomarkers, by sex

Biomarker and FFQ measurement	Male n = 81	Female n = 80
Isoflavones (ug/gUCr)		
Soy protein (g/day)[1]	0.57*	0.32*
Total carotenoids (mg/L plasma)		
Fruit (inc 100% fruit juice) (g/day)[2]	0.16	0.41*
Vegetables (g/day)[2]	0.20	0.24*
Total fruit and vegetables (g/day)[2]	0.22	0.41*
Plasma EPA + DHA (% total FA)		
Fish and seafood (g/day)	0.36*	0.36*
Plasma polyunsaturated FA (% total FA)		
Polyunsaturated fat (%E)	0.08	0.18
Plasma odd chain saturated FA (% total FA)		
Dairy fat (g/day)	0.51*	0.10

EPA, eicosapentaenoic acid; DHA, docosahexaenoic acid; %E, as a percentage of energy intake; FA, fatty acid

Values were transformed using natural logs or square roots before analysis

Values were adjusted for intra-individual variation, age, sex, total fat intake (as a % of energy), and energy intake

[1] Phytoestrogen supplement users were excluded (n = 17).

[2] Carotenoid supplement users were excluded (n = 17).

* Correlation is statistically significant, $P < 0.05$
